# Feasibility of Detecting Fluorescent Marking Clip with Novel Fluorescence Detection System in Minimally Invasive Stomach and Esophageal Surgery

**DOI:** 10.3390/jcm14030717

**Published:** 2025-01-23

**Authors:** Hideyuki Wada, Yuma Ebihara, Hironobu Takano, Mariko Hayashi, Takeo Nitta, Toshiaki Shichinohe, Satoshi Hirano

**Affiliations:** Department of Gastroenterological Surgery II, Hokkaido University Faculty of Medicine Kita 15, Nishi 7, Kita-ku, Sapporo 060-8638, Japan; ajh12427@elms.hokudai.ac.jp (Y.E.); htakano131@huhp.hokudai.ac.jp (H.T.); eep44442@elms.hokudai.ac.jp (M.H.); nitta1016@huhp.hokudai.ac.jp (T.N.); shichino@med.hokudai.ac.jp (T.S.); satto@med.hokudai.ac.jp (S.H.)

**Keywords:** fluorescent clip, fluorescence spectroscopy, minimally invasive surgery

## Abstract

**Background**: Determining the optimal resection line for an organ that cannot be palpated is crucial, but challenging, in minimally invasive gastrointestinal (GI) surgery. Therefore, there is an urgent need to establish the most effective method for tumor localization. We hypothesize that our novel near-infrared (NIR) fluorescence detection system will enable the highly accurate detection of fluorescent clips marking GI cancer. **Methods**: Twenty-five patients with gastric cancer, esophagogastric junctional cancer, or esophageal cancer will be enrolled. NIR fluorescent clips will be placed endoscopically around the tumor on the day before surgery. Patients in whom clip dislodgement is confirmed by preoperative abdominal radiography will be excluded. The clips will be placed before the transection of the organ, and those on the surgical specimen will be observed after transection using both the novel NIR fluorescence detection system and an existing NIR fluorescence imaging system. The detection rate and time, the fluorescence intensity, surgical margins, and adverse events will be evaluated. This study has been registered in the Japan Registry of Clinical Trials, with the code jRCTs012240043. (**Expected**) **Results**: As the novel fluorescence detection system allows for higher-sensitivity detection by analyzing the spectral characteristics of fluorescence and measuring the peak values, we anticipate that this new system will detect the fluorescent clips with high accuracy. **Conclusions**: This study aims to establish a novel tumor-marking method using fluorescent clips and a new detection system that can be easily applied in various medical facilities.

## 1. Introduction

Minimally invasive surgery (MIS) for gastric and esophageal cancer is widely accepted worldwide, and most gastrectomies and esophagectomies are performed entirely under laparoscopic and thoracoscopic guidance, including robotic surgery [1,2,3,4]. Although it is important to determine the precise incision line of the organ intraoperatively for upper gastrointestinal (GI) surgery, it is generally difficult to confirm the location of the tumor without palpation, especially in early-stage cancer, during MIS. The optimal resection of the organ prevents not only non-curative resection with insufficient surgical margins but also troublesome anastomosis involving a highly resected esophagus in the mediastinum [5,6]. Moreover, an unnecessary total gastrectomy or the excessive resection of the stomach wall can result in reduced food intake and postoperative weight loss [7,8].

Although methods such as ink tattooing, endoscopic clipping, and intraoperative endoscopy are currently available to identify tumor locations during surgery, the dissemination of ink into the wall complicates the determination of both the precise incision line of the organ and the dissectable layer of the adjacent tissue [9,10]. Regarding endoscopic clip marking, surgeons may have difficulty detecting it without significant experience using a laparoscopic ultrasound probe due to the small size of the clips. Although intraoperative endoscopy is the most reliable method to identify the location of the tumor, it can lead to increased medical costs and prolonged operation times, owing to the need for securing an endoscopist and using multiple pieces of equipment.

Recent reports describe a tumor-marking method that uses an endoscopic clip labeled with near-infrared (NIR) fluorescence, matching almost the same wavelength as indocyanine green (ICG), alongside an NIR fluorescence imaging system [11,12,13,14,15,16]. While this method enables the accurate and rapid intraoperative identification of the tumor location and resection lines, the clips may sometimes go undetected by the existing imaging system. This problem arises because NIR light can penetrate tissue only up to 5–8 mm, depending on the thickness of the gastric or esophageal wall and the tumor’s location [17]. Even when the clips are detectable, various adjustments are often required, such as placing the clips on the anterior wall regardless of the tumor’s location, expanding the gastric wall with forceps, releasing the omentum, and inverting the stomach to enable observation from the posterior wall. These procedural modifications can also contribute to prolonged operative periods. We developed a novel NIR fluorescence detection system, Lumifinder™ (Advantest Co, Tokyo, Japan), which may address the limitations of the current imaging systems [18]. This system successfully enabled the detection of fluorescent clips in the porcine stomach, which typically has a thicker wall than the human stomach. In this study, we hypothesized that our novel NIR fluorescence detection system would enable the highly accurate detection of fluorescent clips at any location in the stomach and esophagus.

## 2. Materials and Methods

### 2.1. Study Design

This is a single-center, open, interventional, and exploratory prospective clinical trial. This study has been registered in the Japan Registry of Clinical Trials, with the code jRCTs012240043 (https://jrct.niph.go.jp/en-latest-detail/jRCTs012240043) (accessed on 18 October 2024).

**Figure 1:**
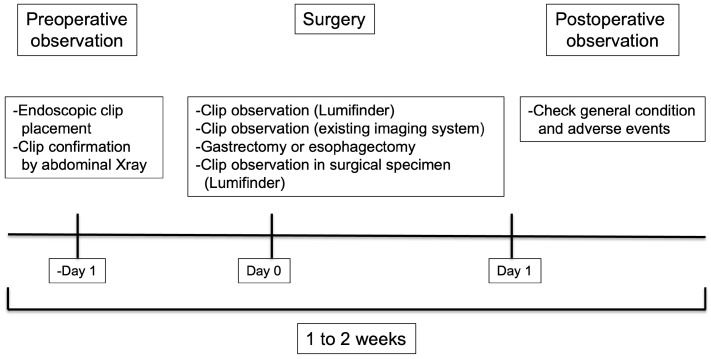
Detailed schedule.

**Figure 2:**
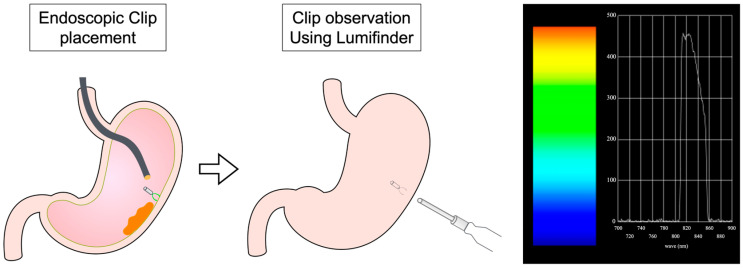
Study schema of endoscopic clip placement and clip observation using Lumifinder™. The spectrum graph is described and recorded on the system when fluorescence is detected. The brown area indicates the tumor. The color gradient next to the fluorescence spectrum graph represents the distribution of colors corresponding to wavelengths.

The detailed schedule and schema are presented in Figure 1 and Figure 2, respectively. All research subjects will be registered until 31 March 2026. The collected data will be analyzed up until 31 March 2027. The data will also be stored and may be utilized in future studies.

#### 2.1.1. Preoperative Procedures

1. NIR fluorescence clips are placed endoscopically by an endoscopist 24 h before surgery, as follows:
  i. Distal gastrectomy, total gastrectomy, and esophagectomy: place two clips on the oral side of the tumor.
  ii. Proximal gastrectomy: place two clips on the oral side and two clips on the anal side of the tumor.
2. An abdominal X-ray is examined to confirm the location of the clips.

#### 2.1.2. Intraoperative Procedures

1. Immediately after initiating laparoscopic, thoracoscopic, or robotic procedures, the surgeon uses the NIR fluorescence detection system to locate the NIR fluorescent clips on the target organ. The probe tip should be positioned 4 cm from the target organ and applied as perpendicularly as possible to the organ in order to excite and detect the fluorescence of the clips. The quantification of the fluorescence of the clips and the measurement of the detection time are recorded.
2. As a reference, the NIR fluorescent clips are examined using an existing NIR fluorescence imaging system. In laparoscopic and thoracoscopic surgery, the endoscope is equipped with a fluorescence observation mode. This endoscope is also used in robotic surgery for fluorescence observation, allowing the study to be conducted under similar conditions.
3. If the fluorescent clip cannot be detected, the endoscopist performs an additional intraoperative oral endoscopy.
4. After these examinations, the gastric cancer, esophagogastric junctional cancer, or esophageal cancer is resected in the usual manner.

#### 2.1.3. Postoperative Procedures

1. The NIR fluorescence of the clips is observed and quantified by the NIR fluorescence detection system in the surgical specimen.
2. During the 1–2 weeks following surgery, the patient’s overall condition and any adverse events are monitored through clinical observation, blood tests, and chest and abdominal X-rays, as appropriate.

### 2.2. Materials and Equipment

#### 2.2.1. Novel Laparoscopic Near-Infrared Fluorescence Spectrum System (Lumifinder™)

**Figure 3:**
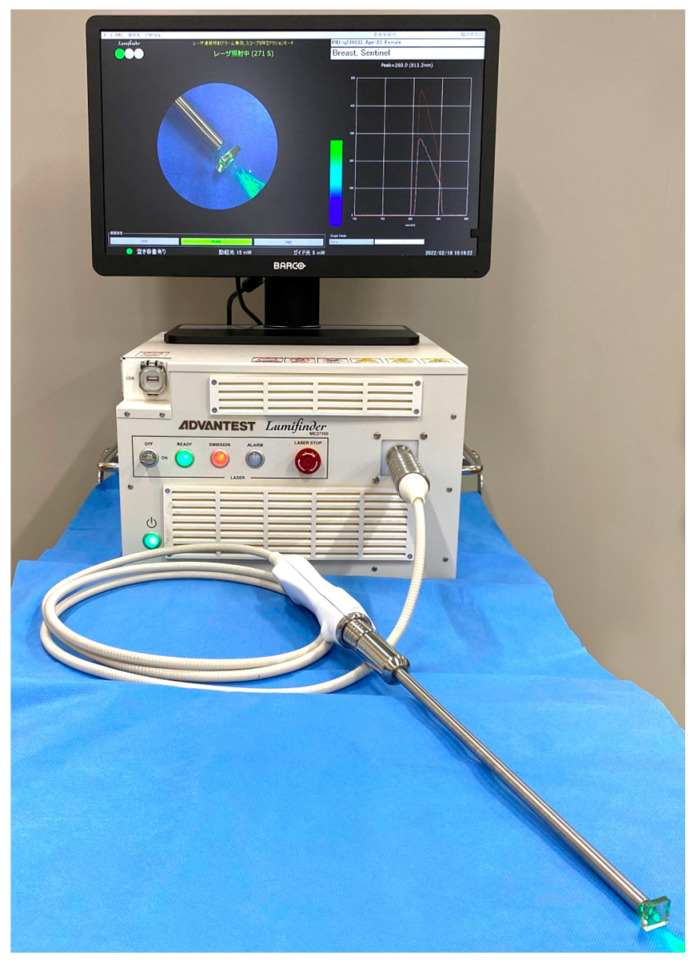
Novel laparoscopic near-infrared fluorescence spectrum system.

The Lumifinder™ has been extensively described in previous studies [18]. The system utilizes a laser diode with a wavelength of 785 nm and a maximum output power of 5 mW as the excitation source. For irradiation, the laser beam is directed onto the central region of a fused-silica coaxial fiber configured in a Y-shape. The near-infrared (NIR) fluorescence emitted by the NIR fluorescent clips is gathered through the outer segment of the coaxial fiber and analyzed using spectroscopy. Since the fluorescence peaks at around 840 nm—close to the excitation wavelength of 785 nm—a narrowband notch filter is placed at the entrance of the spectroscope to suppress signals from the excitation source (Figure 3). The probe (diameter = 10 mm, length = 350 mm) can be inserted through a 12 mm port into either the abdominal or thoracic cavity. The fluorescence of the NIR fluorescent clip is excited by the excitation light source from the probe tip close to the target organ. The wavelength graph is described and recorded on the system when fluorescence is detected.

#### 2.2.2. Endoscopic NIR Fluorescence Imaging System

We will utilize the VISERA ELITE 2 system (Olympus Co., Tokyo, Japan), which activates ICG with emitted light at a wavelength of 760 nm, enabling both intraoperative white light imaging and NIR fluorescence visualization following exploration with Lumifinder™ during laparoscopy, thoracoscopy, and robotic surgery.

#### 2.2.3. The Fluorescent Clip (XEMEX FS Marker^®^)

**Figure 4:**
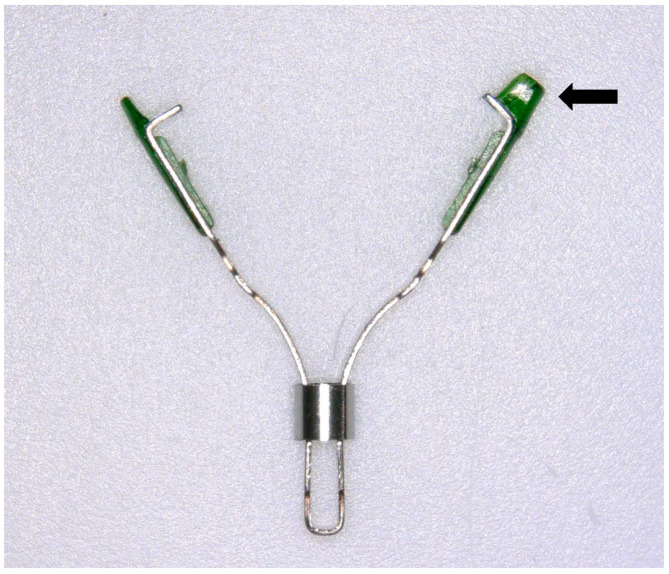
The fluorescent clip. The arrow indicates the near-infrared fluorescent resin applied to the arms.

The XEMEX FS Marker^®^ (Zeon Medical Co., Tokyo, Japan) has near-infrared fluorescent resin applied to its arms, which hold the GI mucosa and absorb and emit the near-infrared fluorescence with peaks of 760 and 790 nm, respectively (Figure 4). This clip has been approved for clinical use (registration number: 30500BZX00292000).

#### 2.2.4. Endpoints

The primary endpoint of this study is to determine whether the Lumifinder™ can detect the XEMEX FS Marker^®^ around the tumor. Secondary endpoints include assessing the variation in signal intensity at different tumor locations, differences in the thickness of the gastric or esophageal wall, the time required for clip identification and fluorescence measurement, and the measurement of the proximal and distal margins of the surgical specimen. The safety endpoint involves evaluating any adverse events associated with the use of Lumifinder™ and the XEMEX FS Marker ^®^.

#### 2.2.5. Study Patients and Eligibility Criteria

This study will include patients diagnosed with gastric, esophagogastric junction, or esophageal cancer who are either visiting or have been admitted to the Department of Gastroenterological Surgery II at Hokkaido University Hospital and are considered eligible for surgery by the department. The eligibility criteria are listed in Table 1. The principal investigator and sub-investigator will provide a detailed explanation of the study to the patients at the Department. If the patients agree to participate, informed consent will be obtained using a dedicated consent form approved by the Research Ethics Board of Hokkaido University Hospital.

#### 2.2.6. Target Number of Patients

Twenty-five patients will be enrolled prospectively. We will recruit participants from 1 February 2025, to 31 March 2026. Given that a total of 50 or more patients meet the eligibility criteria annually in our department, it is anticipated that the target number will be achieved within one year.

#### 2.2.7. The Determination of the Sample Size

The target sample size was set to 25 cases to account for potential clip dislodgement and ineligible cases. This determination was based on calculations using the Wilson’s score method, which indicated that 21 cases would be required to achieve a width of less than 30% for the 95% confidence interval, assuming a success rate of 90% for the fluorescence measurement and the position identification of the XEMEX FS marker using Lumifinder™, with a probability of 80% or higher. This was determined in collaboration with the statistician responsible for the statistical analysis at the Data Science Center, the Institute for Medical and Health Science Research Development, Hokkaido University Hospital.

#### 2.2.8. Statistical Analysis Methods

The patients’ demographic and baseline characteristics will be described using appropriate statistical measures: the mean and the standard deviation or the median and the interquartile range, as needed, for the continuous data and the frequency with the percentage for the categorical data. For the primary endpoint, the success rate and a 95% confidence interval will be calculated using the Wilson’s score method. The secondary endpoints will be analyzed by estimating values with corresponding 95% confidence intervals, and the relationships among the variables will be assessed using visual and statistical tools such as scatter plots, box plots, and Pearson’s correlation coefficients, among others, as appropriate. Cases in which all the clips are confirmed to have been dislodged during preoperative abdominal radiographs or intraoperative evaluations will be excluded from the statistical analysis.

#### 2.2.9. Anticipated Benefits and Disadvantages (Burdens and Risks)

- Anticipated benefits: determining the optimal resection line using fluorescent clip markings may contribute to improved curability and the maintenance of postoperative function, including food intake.
- Anticipated disadvantages: The preoperative oral endoscopic marking and the intraoperative fluorescence measurement performed for research purposes will each involve a time commitment of approximately 30 min. Adverse events such as perforation, bleeding, tissue damage, and infection are mentioned in the package inserts of the Zemex FS Marker and Lumifinder™. However, the likelihood of such adverse events occurring owing to the use of these devices is extremely low, provided that endoscopic procedures and clipping are performed by endoscopy specialists and that the maintenance, cleaning, disinfection, and sterilization of the equipment and scope are conducted properly. If the fluorescent clip cannot be detected or is dislodged during surgery, additional intraoperative oral endoscopy may be required to determine the organ resection line, and the prohibition of MRI imaging after clip marking, as stated in the package insert, could potentially disadvantage the study participants.

#### 2.2.10. Handling of Adverse Events

Adverse events are any unfavorable or unintended injuries or symptoms (including abnormal clinical laboratory values) that occur in a research subject, whether or not they are related to the research that has been conducted, and that are recognized during the observation period from the day before the surgery marking.

When the investigator recognizes an adverse event, they will immediately take appropriate measures and provide treatment. The name of the adverse event, the date of onset and the date of resolution, the severity (mild, moderate, or severe), the outcome, the causal relationship with the study (related, unrelated), and the course of the event will be recorded. In addition, if the research device is discontinued or treatment for the adverse event is required, the research subject will be informed accordingly.

If the doctor in charge of the research becomes aware of the occurrence of any serious adverse event (death, life-threatening conditions, hospitalization or the prolongation of hospitalization, persistent or significant disability/incapacity, and any congenital anomaly in offspring), he/she shall take necessary measures, such as providing an explanation to the research subject and promptly reporting the event to the principal investigator. The principal investigator shall promptly report the event to the administrator of the medical institution of the relevant organization, take appropriate measures, and share information with the sub-investigator.

If an event that meets the conditions for an important adverse event (eczema, nausea, vomiting, etc., associated with fluorescent clip marking) occurs, the principal investigator will promptly report it, following the procedure for reporting serious adverse events. The principal or sub-investigator will appropriately record other adverse events in the medical record. 

#### 2.2.11. The Discontinuation or Termination of the Study

If a subject falls into any of the categories listed below, the principal investigator will promptly discontinue the subject’s participation in the study and take appropriate measures. Additionally, the investigator will monitor any adverse events identified during discontinuation and ensure the subject’s safety by performing necessary examinations and tests and providing appropriate medical care, contingent on the subject’s cooperation. The examinations conducted during discontinuation will be limited to those deemed medically relevant, respecting the subject’s autonomy and informed consent. Data relevant to the study will be collected promptly upon discontinuing the subject’s participation. In cases where a subject ceases to attend scheduled visits during the research period, the principal investigator will attempt to contact the subject via telephone or other means to encourage them to return for follow-up. If the subject cannot return to the hospital, their health condition will be assessed remotely through verbal communication. Any adverse events will be managed according to the relevant section’s protocol. The date and reason for discontinuation will be recorded in the Electronic Data Capture (EDC) system. The date of discontinuation will be defined as the date on which the principal investigator decides to terminate the subject’s participation in the study. The following are the criteria for discontinuation:

1. Subject requests treatment modification or discontinuation—to uphold ethical considerations and respect for the subject’s autonomy.
2. Observation of an adverse event that precludes continued participation—based on safety concerns.
3. Occurrence of death or a life-threatening condition—to prioritize subject safety.
4. Worsening of the primary disease—for the subject’s safety and well-being.
5. Discovery of pregnancy—to mitigate the risks associated with potential harm to the subject or fetus.
6. Deviation from the selection criteria or the violation of the exclusion criteria—to maintain the study’s integrity and validity.
7. Failure to attend scheduled hospital visits—when continued participation cannot be assured.
8. Confirmation of clip displacement via preoperative abdominal X-ray or intraoperative observation—rendering study continuation unfeasible.
9. Other reasons deemed inappropriate by the principal investigator—based on professional judgment.

Criterion 1 was established to address ethical considerations, while criteria 2–5 and 6–9 were established to ensure subject safety and accommodate practical difficulties in continuing the study, respectively.

#### 2.2.12. The Discontinuation of the Entire Study

If any of the following situations arise, the principal investigator (PI) of the study shall consider suspending the entire study. If a suspension decision is made, the PI shall promptly notify all the responsible investigators in writing, including the reasons for the suspension. Furthermore, the PI shall submit a notice of the suspension of the entire study to the Certified Clinical Research Review Board (CRB) and the Minister of Health, Labour, and Welfare using the prescribed format within 10 days.

The PI shall promptly notify the administrators of the implementing medical institutions, as well as the research participants currently involved in the study. Additionally, appropriate examinations or interventions to confirm the safety of the research participants shall be conducted.

1. In cases where information is obtained that compromises, or may compromise, the ethical validity or the scientific rationality of the study that may affect the continuation of the study.
2. In cases where information is obtained that compromises, or may compromise, the propriety of the study’s implementation or the reliability of the study’s results.
3. In cases where it is determined that the anticipated risks of the study outweigh the expected benefits, or where information suggests that sufficient results have been achieved or will not be achieved through the study.
4. In cases where significant information regarding the quality, safety, or efficacy of the research equipment is obtained, leading to the conclusion that the continuation of the entire study is not feasible.

If the study is suspended for any of the following reasons within the implementing medical institutions, the PI shall promptly notify the participating research subjects and conduct appropriate examinations or interventions to confirm their safety:

1. If the Certified Clinical Research Review Board issues a recommendation or directive to suspend the study.
2. If the Certified Clinical Research Review Board issues a directive to modify the study plan and it is deemed challenging to accept said modification.

#### 2.2.13. Termination of Research

The study is considered complete when the following tasks are fully accomplished:

1. The completion of subject enrollment and observation periods.
2. The preparation of the primary endpoint report, the final study report, and the summary of the final report.
3. The submission of the primary endpoint report to the Ministry of Health, Labour, and Welfare.
4. The submission of a summary of the final report, study protocol, and statistical analysis plan to the Ministry of Health, Labour, and Welfare.
5. The submission of the primary endpoint report, the final report, and the summary of the final report to the administrators of the participating medical institutions.
6. The registration of the summary of the study results in the jRCT.
7. The notification of the administrators of the participating institutions regarding the publication of the study results.

#### 2.2.14. Reporting of Protocol Deviations

When the principal investigator becomes aware that the study is not in compliance with the Clinical Research Act (including enforcement regulations and related notifications) or the research protocol (hereinafter referred to as “non-compliance”), they shall promptly report this to the Director of Hokkaido University Hospital. If a sub-investigator identifies a protocol deviation, they shall promptly report it to the principal investigator.

If the principal investigator determines that a deviation is particularly serious, they must immediately report it to the certified clinical research review committee. Additionally, they must implement measures to prevent recurrence and ensure future compliance.

In the event of a change in the principal investigator, the research and implementation plans must be revised. This revision requires a review by the certified clinical research review committee and that the Minister of Health, Labour, and Welfare be notified of the implementation plan. Consequently, completing these procedures may take a certain amount of time. Furthermore, in some instances, the change may not be announced until just before it takes effect, making it challenging to finalize the procedural requirements in advance.

Therefore, even if the principal investigator is temporarily absent due to the change, the study does not constitute a “deviation” if the research management system is maintained by the sub-investigators and the medical care system for enrolled patients is ensured.

#### 2.2.15. Post-Research Actions

After the completion of this study, the medical care considered most appropriate for the subjects will be provided, incorporating the findings obtained from this research.

#### 2.2.16. Data Collection

In this study, the EDC system will be used to record the necessary information. The principal investigator and the administrator of the medical institution where the study will be conducted will ensure that the data in the case report forms and all other reports are accurate and complete.

#### 2.2.17. Case Report Form Creation (EDC System Input) and Storage Management

The principal investigator will prepare a “case report form entry guide” that explains the entry procedures and notes on completion, etc., if necessary. The person completing the case report form will enter the data into the EDC system in accordance with the “case report form entry guide”. Hereafter, case report form completion will be referred to as EDC system entry.

The investigator will enter data into the EDC system as the study progresses for all the subjects enrolled until the study is completed.

When entering data into the EDC system, a number (subject identification) that is not related to the personal information of the research subject will be assigned, and a correspondence table with the registration numbers will be created so that the data and individuals can be identified. Still, this correspondence table will not be provided and will be kept at the medical institution where the study is conducted. It will not contain any information that could identify the research subjects.

#### 2.2.18. Monitoring

The monitoring of this research shall be carried out in accordance with the Monitoring Procedures. In addition, the principal investigator and the institution implementing the study will ensure that all the records related to the research subjects are readily accessible for direct review upon request by the monitoring personnel. The monitoring personnel shall confirm that the research is being conducted in adherence to the Clinical Research Act, applicable notifications, and the approved research protocol.

## 3. Expected Results and Discussion

Fluorescent clip-based tumor marking is one of the most promising methods for intraoperative identification during GI cancer surgery. However, Kumagai et al. reported that in fluorescent clip-based tumor marking using the current imaging system for gastric cancer surgery, the detection rate of the fluorescent clips was only 75%, which is significantly lower than the identification rate of tumors and clips (100%) using intraoperative endoscopy [16]. The primary cause of this low detection rate is thought to be the limitation of the current NIR fluorescent imaging system, which can only observe tissue thicknesses of 5–10 mm, whereas the gastric wall can sometimes be over 10 mm thick [17,19,20].

Our newly developed fluorescence detection system, “Lumifinder™”, enables the higher-sensitivity detection of fluorescence by analyzing the spectral characteristics of fluorescence and measuring peak values. In fact, it has successfully detected fluorescent clips in a porcine stomach with a thickness of 13 mm [18], and its usefulness has been reported in several clinical trials [21,22]. We expect that this new system will be able to detect fluorescent clips with a high probability, regardless of the thickness of the organ wall or the lesion’s location. This method will enable the determination of resection lines that balance curability and functionality, even in facilities where intraoperative endoscopy is challenging. In certain cases, fluorescent clips may not be detectable because of their inherently low fluorescence intensity. Given our extensive synthesis of noble NIR fluorophores and the reported outcomes of their clinical performance in preclinical settings [23], we are confident of our ability to develop a novel fluorescent clip with a significantly enhanced fluorescence intensity in future studies.

However, this ideal marking method has certain drawbacks. Because our spectral system does not objectively display fluorescent signals like an imaging system, it requires “exploration” to locate the fluorescent clips. As a result, detecting the signals may take longer than with traditional imaging systems in some cases. In this study, observations using the existing near-infrared fluorescence imaging systems are also planned as a reference. However, a prospective comparative study between our new fluorescence detection system and the near-infrared fluorescence imaging system will be necessary to determine the future standard of care.

Additionally, distinguishing whether the clip is on the anterior or the posterior wall is challenging. To compensate for these limitations, thorough preoperative imaging is necessary to confirm the lesions.

## 4. Conclusions

This study will be conducted with the aim of establishing a novel tumor-marking method utilizing fluorescent clips and an innovative fluorescence detection system. This approach is more reliable, simpler, and less invasive than the existing methods and can be implemented in a wide range of medical facilities. Therefore, it is expected to become the standard technique for minimally invasive GI surgery.

## Figures and Tables

**Table 1:** Eligibility criteria of the study.

Inclusion Criteria	Exclusion Criteria
(i) Patients aged 18 years or older at the time of obtaining informed consent.	(i) Patients under 18 years of age at the time of consent.
(ii) Patients with a preoperative diagnosis of primary gastric cancer, esophagogastric junctional cancer, or esophageal cancer (including patients who have received neoadjuvant chemo/chemoradiotherapy).	(ii) Patients with a history of gastrectomy or esophagectomy.
(iii) Patients scheduled to undergo laparoscopic or thoracoscopic surgery (including robot-assisted surgery) under general anesthesia.	(iii) Patients deemed unsuitable for laparoscopic or thoracoscopic surgery due to a history of upper abdominal surgery or other factors.
(iv) Patients who have voluntarily provided written informed consent after receiving a thorough explanation of the study and demonstrating a full understanding of its contents.	(iv) Patients with a history of metal allergies.
	(v) Patients considered unsuitable for participation in the study by the principal investigator or sub-investigators.

## Data Availability

The data underlying this article will be shared upon reasonable request to the corresponding author.
